# Positive interaction between GPER and β-alanine in the dorsal root ganglion uncovers potential mechanisms: mediating continuous neuronal sensitization and neuroinflammation responses in neuropathic pain

**DOI:** 10.1186/s12974-022-02524-9

**Published:** 2022-06-21

**Authors:** Zhenzhen Xu, Wanli Xie, Yiqi Feng, Yanting Wang, Xia Li, Jie Liu, Yue Xiong, Yuyao He, Lu Chen, Guoyang Liu, Qingping Wu

**Affiliations:** Department of Anesthesiology, Union Hospital, Tongji Medical College, Huazhong University of Science and Technology, Wuhan, 430022 China

**Keywords:** G protein-coupled oestrogen receptor, β-Alanine, Dorsal root ganglion, Neuroinflammation, Neuropathic pain

## Abstract

**Background:**

The pathogenesis of neuropathic pain and the reasons for the prolonged unhealing remain unknown. Increasing evidence suggests that sex oestrogen differences play a role in pain sensitivity, but few studies have focused on the oestrogen receptor which may be an important molecular component contributing to peripheral pain transduction. We aimed to investigate the impact of oestrogen receptors on the nociceptive neuronal response in the dorsal root ganglion (DRG) and spinal dorsal horn using a spared nerve injury (SNI) rat model of chronic pain.

**Methods:**

We intrathecally (i.t.) administered a class of oestrogen receptor antagonists and agonists intrathecal (i.t.) administrated to male rats with SNI or normal rats to identify the main receptor. Moreover, we assessed genes identified through genomic metabolic analysis to determine the key metabolism point and elucidate potential mechanisms mediating continuous neuronal sensitization and neuroinflammatory responses in neuropathic pain. The excitability of DRG neurons was detected using the patch-clamp technique. Primary culture was used to extract microglia and DRG neurons, and siRNA transfection was used to silence receptor protein expression. Immunofluorescence, Western blotting, RT-PCR and behavioural testing were used to assess the expression, cellular distribution, and actions of the main receptor and its related signalling molecules.

**Results:**

Increasing the expression and function of G protein-coupled oestrogen receptor (GPER), but not oestrogen receptor-α (ERα) and oestrogen receptor-β (ERβ), in the DRG neuron and microglia, but not the dorsal spinal cord, contributed to SNI-induced neuronal sensitization. Inhibiting GPER expression in the DRG alleviated SNI-induced pain behaviours and neuroinflammation by simultaneously downregulating iNOS, IL-1β and IL-6 expression and restoring GABAα2 expression. Additionally, the positive interaction between GPER and β-alanine and subsequent β-alanine accumulation enhances pain sensation and promotes chronic pain development.

**Conclusion:**

GPER activation in the DRG induces a positive association between β-alanine with iNOS, IL-1β and IL-6 expression and represses GABAα2 involved in post-SNI neuropathic pain development. Blocking GPER and eliminating β-alanine in the DRG neurons and microglia may prevent neuropathic pain development.

**Supplementary Information:**

The online version contains supplementary material available at 10.1186/s12974-022-02524-9.

## Introduction

There is increasing evidence of the critical roles of oestrogen in the peripheral and central nervous systems [1–3]. Oestrogen targets neurons to regulate neural development, plasticity, and neuroprotective actions in behavioural and cognitive functions [4]. Oestrogen exerts its effects through its receptors, which are broadly distributed in the nervous system [5, 6]. However, studies on the role of oestrogen receptors (ERs), which may be crucially involved in peripheral pain transduction, are limited. It remains unclear whether modulating ERs could represent an effective therapeutic strategy for neuropathic pain. Anatomical studies have confirmed that pain perception results from nociceptive signals produced by nociceptive neuron activation in the peripheral sensory nerve [7]. Subsequently, the nociceptive signals are transduced via the dorsal root ganglion (DRG) neurons, which synapse to spinal dorsal horn (SDH) neurons and finally project to the thalamus and cerebral cortex [8]. Neuropathic pain is a clinically common chronic refractory pain syndrome with a global prevalence of approximately 7–10% [9]. Neuropathic pain has a complex pathogenesis with an indeterminate molecular mechanism, which impedes its clinical treatment. Recent studies have demonstrated increased intracellular Cl^−^ levels in the DRG neurons after sciatic nerve section or inflammation [10–12]. There has been extensive research on chloride channels in primary sensory neurons given that chloride channel activation in sensory neurons may cause chloride efflux and depolarization due to high intracellular chloride concentrations [13, 14]. Anion channels, especially chloride channels, may be involved in neuropathic pain pathogenesis [15]. Numerous studies have investigated GABAα2-mediated analgesia in neuropathic pain, given that the absence of GABAα2 is a classic neuropathic pain sign accompanied by neuroinflammation [16, 17]. Spared nerve injury (SNI) is the most recognized method for simulating neuropathic pain [18, 19]. Numerous studies on the pathogenesis, prevention, and treatment of neuropathic pain have suggested that the pain threshold is sex-specific [20, 21]. Notably, ERs are present in the DRG and SDH. Few studies have investigated pain regulation by oestrogen or ERs through ion channels. GPER is a potential 17β-oestrogen target that is modulated by the ERs in breast cancer cells [22]. Furthermore, oestradiol modulates the efficacy of synaptic inhibition by decreasing the dwell time of GABA_A_ receptors at inhibitory synapses [23]; however, the receptors involved remain unclear. The possible function of ERs in pain modulation is further supported by the ubiquitous expression of ERs in nociceptive system neurons [24]. No studies have focused on the effects of oestrogen on pain modulation or the role of ERs in mediating continuous neuronal sensitization and neuroinflammation responses in neuropathic pain. Additionally, oestrogen can exert its effects in the absence of ERs. This study aimed to identify the main ERs involved in neuropathic pain and determine their related genomics and metabolomics to explore the role of ER-related metabolites in neuropathic pain. Specifically, we aimed to examine the metabolic mechanism of ERs in pain and elucidate the potential relationship of ERs with neuronal sensitivity and neuroinflammation.

## Methods

### Animals

We purchased adult male Sprague-Dawley rats (8–10 weeks old, 180–200 g, *n* = 400, Additional file 2: Table S1) from the Animal Centre of the Charles River (Charles River Laboratories, US). The use of animals was approved by The Institutional Animal Care and Use Committee at Tongji Medical College, Huazhong University of Science and Technology, China. The animals were housed in plastic boxes with controlled temperature (24 ± 2 °C), humidity (40–50%), and a 12:12 h light: dark cycle. We selected rats with relatively uniform and stable baseline responses to cold, mechanical, and hot stimuli. All protocols were approved by the Animal Ethics Committee of the Tongji Medical College, Huazhong University of Science and Technology (approval No. 2405) on December 30, 2020. Further, they were consistent with the Guidelines for the Care and Use of Laboratory Animals, published by the US National Institutes of Health.

### Surgical procedure for establishing a neuropathic pain model through spared nerve injury (SNI)

We established a model of neuropathic pain using SNI as previously reported [25]. Experimental procedures were performed on animals under anaesthesia through intraperitoneal administration of 40 mg/kg sodium pentobarbital (Sigma-Aldrich, St. Louis, MO, USA). Care was exercised to prevent infection and reduce the inflammation impact. The skin was cut to expose the sciatic nerve and its three terminal branches directly through the part formed by the biceps muscle: the lateral side, common fibular nerve, and tibial nerves. The tibial and common peroneal nerves were cut and ligated through SNI, while the sural nerve was preserved. Given that the common peroneal and tibial nerves are closely connected, we subsequently removed 3–5 mm of the distal nerve ends. Care was taken to avoid damaging the nearby sural nerve. All wounds were postoperatively irrigated with sterile saline and closed in layers. Experimenters blinded to the treatments performed the behavioural tests, electrophysiologic recordings, and Western blot and immunohistochemical experiments. The primary outcome was the effect of pharmacologic and genetic interventions involving different ERs in the DRG and SDH on pain-related aversion. We did not use analgesics.

### Groups and drug intervention

All male rats were randomly divided into groups containing 6–8 rats. Drugs were dissolved in 1% DMSO and injected through a catheter for 24 h. Intrathecal catheters were implanted on SNI day 5 as previously described [26]. We purchased 1,3-Bis (4-hydroxyphenyl)-4-methyl-5-[4-(2-piperidinyl ethoxy) phenol]-1*H*-pyrazole dihydrochloride (MPP) and PHTPP from Tocris Bioscience (United Kingdom). We purchased (3aS*,4R*,9bR*)-4-(6-Bromo-1,3-benzodioxol-5-yl)-3a,4,5,9b-3H-cyclopenta [c] quinolone (G15); 4,4ʹ,4ʹʹ-(4-propyl-[1*H*]-pyrazole-1,3,5-triyl) trisphenol (PPT); 2,3-bis (4-hydroxyphenyl)-propionitrile (DPN); (±)-1-[(3aR*,4S*, 9bS*)-4-(6-bromo-1,3-benzodioxol-5-yl)-3a,4,5,9b-tetrahydro-3*H*-cyclopenta [c]quinolin-8-yl]-ethanone; and dimethyl sulfoxide from Sigma (USA). MPP (1 mM), G15 (1 mM), PPT (1 mM), DPN (5 mM), and G1 (5 mM) were dissolved in dimethyl sulfoxide. β-Alanine (10 mM) was dissolved in saline. Stock solutions of the drugs were diluted in normal saline or artificial cerebrospinal fluid. Briefly, a saline-filled sterile catheter was inserted through the intervertebral space at L_5_/L_6_; moreover, the tube tip was positioned at the lumbosacral spinal level. We excluded animals with postoperative hindlimb paralysis or paresis. Animals without movement disorders received 2% lidocaine through the catheter to verify the intraspinal location. Correct catheterization was confirmed by immediate bilateral hindlimb paralysis (within 15 s) lasting 20–30 min. We excluded animals that lacked these features. DRGs for patch-clamp experiments were incubated with G1, G15, and β-alanine in vitro.

### Measurement of β-alanine

BioVision’s β-Alanine Assay Kit allows simple and sensitive detection of β-alanine. In the kit, β-alanine is converted to pyruvate, which is specifically detected based on proportional colour (*λ* = 570 nm: 0–10 nmol) or fluorescence (Ex/Em 535/587 nm: 01 nmol) development. The serum β-alanine levels are ~ 2476 μg/ml (~ 3–9 nmol/10 μl). Briefly, tissues or cells (1 × 10^6^) were homogenized in a 100 μl Assay Buffer centrifuge to remove insoluble material at 13,000*g* for 10 min. Subsequently, we directly diluted 10–50 μl of deproteinized serum samples in the Assay Buffer and added them to a 96-well plate at 50 μl/well. Next, sufficient reagents were prepared according to the number of assays to be performed. We added 50 μl reaction mix [containing assay buffer (44 μl), β-alanine converting enzyme (2 μl), β-alanine development mix (2 μl), and β-alanine probe (2 μl)] to each well containing β-alanine standard, test, and background control samples. They were mixed well and incubated for 60 min at 37 °C with protection from light. Subsequently, the optical density was measured at 570 nm in a microplate reader or fluorescence using Ex/Em 535/587 nm. Correction of the background was performed by subtracting the value derived from the β-alanine control sample from all the sample readings. This is because the background reading can be significant and must be subtracted from all sample readings. Finally, we plotted the β-alanine standard curve and calculated the β-alanine levels as follows: *C* = Sa/Sv nmol/μl or mM.

### Behavioural assays

#### Mechanical allodynia

Mechanical allodynia was assessed by measuring the paw withdrawal threshold in response to a calibrated series of von Frey hairs (North coast, USA) [27]. The rats were individually placed in cages with a wire-mesh bottom. A series of calibrated von Frey hairs were applied to the plantar surface of the hind paw in an ascending order 0.4 to 15.0 g with sufficient force to bend the hair for 2 s or until paw withdrawal. Withdrawal responses were considered valid only if the hind paw was completely removed from the customized platform. Each hair was applied five times; moreover, the minimum value causing at least three responses was recorded as the paw withdrawal mechanical threshold (PWMT).

#### Heat hyperalgesia (hot plate test)

Thermal hyperalgesia was assessed as previously described [28]. The thermal withdrawal latency after radiant heat stimulation was measured using an analgesia meter (Ugo Basile, Stoelting, IL, USA). Animals were placed in the chamber and allowed to acclimatize for 30 min before testing. Subsequently, a radiant heat source was focused under the glass floor beneath the hind paws. We adjusted the thermal-stimulus intensity to obtain a baseline thermal withdrawal latency of approximately 20 s. The digital timer automatically recorded the duration between stimulus initiation and the thermal withdrawal latency, with a 30-s cut-off being applied to prevent tissue damage. Each rat was tested at 5-min intervals, and the paw withdrawal thermal latency (PWTL) was calculated as the average of six trials.

#### Cold allodynia (acetone drop method)

Cold sensitivity was measured by applying an acetone drop to the plantar surface of the hind paw as previously described [29, 30]. Rats were housed and habituated for 30 min in transparent plastic boxes using a wire-mesh floor. After the adaptation period, we gently applied acetone against the plantar skin of the left hind paws with an acetone bubble formed with a 0.1-ml syringe alternately three times to hind paws at 5-min intervals. Moreover, we recorded the duration of licking/biting and remaining in the air. Each rat was tested at 5-min intervals, and the paw withdrawal cold duration (PWCD) was calculated as the average of six trials. The itching behavioural test is similar to the cold pain test and records the number of scratches.

### Sample preparation

At predetermined time points after behavioural testing, the animals were deeply anaesthetized using sodium pentobarbital (40 mg/kg, intraperitoneal; Sigma) and killed. Subsequently, ipsilateral L_4-6_ DRGs and SDH tissues were collected. Samples for reverse transcription-polymerase chain reaction (RT-PCR) and western blot experiments were snap-frozen in liquid nitrogen and stored at − 80 °C. We perfused samples used for immunofluorescence imaging through the ascending aorta with saline, followed by 4% paraformaldehyde in 0.1 M phosphate buffer (4 °C, pH 7.4), as previously reported [31].

### Immunofluorescence

The L_4–6_ DRG on the surgical side was removed and fixed in 4% paraformaldehyde overnight, followed by dehydration in 20%/30% sucrose in phosphate buffer at 4 °C. The tissue was cut into 5-μm thick sections with a cryostat (Leica CM1950, Nussloch, Germany). The sections were blocked using 20% bovine serum albumin for 1 h in a 37 °C incubator (303-0S; Beijing Ever Bright Medical Treatment Instrument Co., Ltd., Beijing, China), washed using phosphate-buffered saline (PBS), and incubated with primary antibody (rabbit anti-GPER polyclonal antibody; 1:100, 260,033, Abcam, rabbit anti-ERα polyclonal antibody; 1:100, ab32063; Abcam, rabbit anti-ERβ polyclonal antibody; 1:100, ab187291; Abcam) overnight at 4 °C. After washing with PBS, sections were incubated with secondary antibody (TRITC-conjugated anti-rabbit secondary antibody; 1:100; Santa Cruz Biotechnology, Heidelberg, Germany) for 1 h at 37 °C. For double immunofluorescence staining, we incubated tissue sections with a mixture of anti-GPER antibody and antibodies against neurofilament-200 (NF-200, a marker for myelinated A-fibres, 1:100, ab82259; Abcam, Cambridge, UK), calcitonin gene-related peptide (CGRP, a marker of peptidergic C-type neurons, 1:100; ab81887; Abcam), Neurofilament (a marker for neuron 1:100, ab207176; Abcam, Cambridge, UK); GFAP (GA5) (a marker for astrocytes 1:100, #3670, CST), Anti-Iba1[EPR16589] -IgG1 (a marker for microglia, ab283319; Abcam) for two nights at 4 °C or IB4 (FITC-conjugated; a marker for nonpeptidergic C-type neurons, 5 μg/ml; L2895; Sigma). Except for IB4-treated tissue sections, the other sections were treated using a mixture of FITC- and TRITC-conjugated secondary antibodies at a 1:100 dilution for 1 h at 37 °C. IB4 was 1:750 mixed using TRITC-conjugated secondary antibody. Sections were rinsed using 0.01 M PBS three times, mounted on gelatin-coated slides, and air dried. We visualized immunoreactivity using fluorescence microscopy; further, a negative control was used by omitting the primary antibody to confirm the immunoreaction specificity. Sections were observed at 200 × magnification using a confocal laser scanning microscope (LSM710; Carl Zeiss AG, Oberkochen, Germany). Optical density measurements and data analysis of GPER-positive cells for both types of DRG cells were performed using Image-Pro Plus 6.0 (Media Cybernetics, MD, USA). We recorded the percentage fluorescence results of the positive neurons of the three independent experiments.

### Western blot analysis

We homogenized the frozen tissues and extracted the proteins using a nucleoprotein and cytoplasmic protein extraction kit (Keygen Biotech, Nanjing, China); further, 30 μg of protein was mixed using sodium dodecyl sulphate sample buffer. Proteins were separated using standard sodium dodecyl sulphate–polyacrylamide gel electrophoresis (8–10% gel) and transferred onto 0.45-μm nitrocellulose membranes (Invitrogen, Carlsbad, CA, USA). We blocked membranes in 5% milk for 1 h and incubated them overnight at 4 °C using the following primary antibodies: rabbit anti-GPER (polyclonal antibody; 1:1000, 260,033, Abcam), rabbit anti-ERα (polyclonal antibody; 1:1000, ab32063; Abcam), rabbit anti-ERβ (polyclonal antibody; 1:1000, ab187291; Abcam), rabbit anti-IL-6 (polyclonal antibody; 1:500, A2447; ABclonal), rabbit anti-IL-1β (polyclonal antibody; 1:500, A1112; ABclonal), rabbit anti- GABRα2 (polyclonal antibody; 1:100, A14185; ABclonal)and anti-β-actin (1:1000 dilution, ab8226, Abcam). The next day, we rinsed the membranes using tris-buffered saline Tween thrice for 10 min and incubated them using secondary antibodies (anti- rabbit immunoglobulin G against the primary antibodies). Staining was visualized using enhanced chemiluminescence (GE Healthcare, Chicago, IL, USA). We quantified band intensities using Image J software (Rawak Software Inc., Germany). To determine the interaction between β-alanine and GPER, we used thermostability experiments to illustrate that protein lysates were pre-warmed at a range of temperatures from 42 to 72 °C for 3 min, and incubated in blank, saline solvent group, β-alanine group, respectively, then subjected to western blotting, and the protein expression reduction shift curve was plotted, as previously described [32]. If the displacement curve shifts to the right, it indicates that alanine can be combined with GPER, thereby enhancing the thermal stability of GPER. The four-parameter fitting curve drawing operation is performed in Prism 9.0.

### Quantitative RT-PCR analysis

Total RNA was extracted from the ipsilateral L_4-6_ DRGs of rats using Trizol (Invitrogen) and reverse-transcribed to cDNA using a qRT- PCR kit (Invitrogen) following the manufacturer's instructions [33]. For each cDNA target, 2-μl aliquots of each complete reverse transcriptase reaction were amplified in a 20-μl reaction volume using SYBR Green Real Time PCR Master Mix (Toyobo Co. Ltd., Osaka, Japan) within 45 cycles of 95 °C and 60 °C for 12 s and 35 s, respectively. Amplification was performed using the following primers (Additional file 3: Table S2): GPER, 5ʹ-CAAGCAGTCTTTCCGTCATGC-3′ (forward) and 5′-CTGCTCCGTGCTGTCTGGTAT-3′ (reverse); ERα, 5′-AGATCTTTGACATGTTGCTGGC-3′ (forward) and 5′-CTCGGTGGATGTGGTCCTTC-3′ (reverse); ERβ, 5′-TGCAGCTCAACAGAGGACAGT-3′ (forward) and 5′-TAGAACTTGGCATTCGGTGG-3′ (reverse); GABRA1, 5′-GTGCGACCATAGAACCGAAAG-3′ (forward) and 5′-AAGCGATTCTCAGTGCAGAGG-3′ (reverse); GABRA2, 5′-GTTTATCGCTGTTTGTTACGCG-3′ (forward); and 5′-TGTTCTGTATCATGACGGAGCC-3′ (reverse); GABRA3, 5′-CTTCACCAAGCGAAGTTGGG-3′ (forward) and 5′-GAGTTGAAGAAGCACTGGGAGC-3′ (reverse); GABRA5, 5′-GCACAACATGACGACACCCA-3′ (forward) and 5′-CAGACTTGGTGGAACCATTGG-3′ (reverse); GABRB2, 5′-CCGTATGATTCGACTGCATCC-3′ (forward) and 5′-GCTTTCGATCTCCAACGTGC-3′ (reverse); GABRB3, 5′-TTCGTCTTTGTATTCCTGGCAC-3′ (forward) and 5′-GTGAACATCCATCGGTGCTAGT-3′ (reverse); β-action, 5′-AGCAGATGTGGATCAGCAAG-3′ (forward) and 5′-AACAGTCCGCCTAGAAGCAT-3′ (reverse), species specificity of all primers are of rat origin. We used mRNA β-actin levels as an internal control; further, we obtained a standard curve to determine the relative levels of each cDNA target. We calculated the relative gene expression levels using the 2^−(ΔΔCt)^ method. Triplicate analysis was performed for the expression level of each gene.

### Isolation and primary culture of DRG neurons and microglia

DRG neurons obtained from SD rats (60–80 g) as previously described [34]. Briefly, L4–L6 DRGs were excised in ice-cold DMEM/F12 medium (10565018, GIBCO, USA) and mechanically dissociated from Sham, SNI, SNI + G15, G1, β-alanine groups. After digested with tyrpsin (T9201, Sigma, USA) and collagenase (C9891, Sigma, USA) for 30 min in 37 °C, DRG neurons were seeded onto cover slips coated with poly-l-lysine (P7890, Sigma, USA) in a humidified atmosphere (5% CO_2_, 37 °C) for up to 4 h and then were used for patch-clamp recording.

#### Primary neuron culture and treatment

Primary microglia cultures were prepared from postnatal day 1 (P1) SD pups as previously described. Briefly, SD pup DRGs were harvested and transferred to neurobasal medium (Thermo Fisher) supplemented with B-27 (Thermo Fisher) and penicillin/streptomycin (Thermo Fisher). DRGs were enzymatically dissociated by incubating in 2 ml of HEPES-buffered saline (Sigma) containing collagenase A (1 mg/kg, Sigma) and dispase II (2.4 U/ml, Roche Applied Sciences) for 20 min at 37 °C. Supernatant was carefully removed, replaced with 2 ml of fresh collagenase A/dispase II solution and incubated for 20 min at 37 °C again. Cells were transferred to a tube containing 10 ml of DMEM/10% FBS (Thermo Fisher), centrifuged for 1 min at 200 g at 4 °C, and resuspended in 800 μl of DMEM/10% FBS containing DNase I (150 U/ml, Thermo Fisher). DRG cells were dissociated with fire-polished glass Pasteur pipettes (VWR International) with decreasing tip diameters to create single-cell suspensions. Cells were resuspended in 2 ml of neurobasal medium (Life Technologies), and then centrifuged (260*g*, 10 min) after overlaying on a 10% bovine serum albumin (BSA) gradient (diluted in Neurobasal medium from a 30% BSA solution in PBS, Sigma). Supernatant was removed and resulting pellet resuspended in neurobasal medium for plating. half of the medium was replaced with fresh media every two days.

#### Primary microglia culture and treatment

Primary microglia cultures were prepared from postnatal day 1 (P1) SD pups as previously described [35]. Briefly, SD pup DRGs were harvested, and then placed in DRG supplemented with 10% heat-inactivated FBS, 50 IU/ml penicillin, 50 g/ml streptomycin, 2 mM l-glutamine, 100 M non-essential amino acids. DMEM-F12 and 2 mM sodium pyruvate. DRG tissues were then incubated in 0.25% trypsin–EDTA for 30 min with gentle agitation. The trypsin reaction was stopped by adding a double volume of DMEM/F12 complete medium, and then the DRG tissue was washed 3 times. The tissue is then gently triturated to prepare a single-cell suspension, which is then passed through a 70 m nylon mesh cell strainer to remove tissue debris and aggregates. Cell suspensions were then made in DMEM/F12 complete medium and seeded into T-75 flasks, which were incubated at 37 °C in a humidified 5% CO2. The medium was changed after 5–6 days, and the mixed glial cells were grown to confluence. Microglia were isolated to 97% purity from confluent mixed glial cultures by differential adhesion and magnetic separation, and then allowed to recover 48 h after plating. Treat primary microglia in DMEM/F12 complete medium containing 2% FBS.

### Construction and transfection of small interfering RNA (siRNA)

The GPER-siRNA was designed by Shanghai GeneChem, Co., Ltd., China. To generate expressing siRNA specific for the GPER gene (NM_133573), the RNA interference sequence for the rat GPER (CACAAGAUGUUGGCGUAGA tt; UCUACGCCAACAUCUUGUG tt) and the negative control sequence (UUCUCCGAACGUGUCACGU tt; ACGUGACACGUUCGGAGAA tt) were cloned into the vectors. To infect neuron and microglia, si-RNA particles were added to the culture medium at a multiplicity of infection of 50 nM. The cells were divided into a control, β-alanine, NC-siRNA + β-alanine, GPER-siRNA + β-alanine group. In the NC-siRNA + β-alanine group, we treated the cells with NC-siRNA, which did not link with target gene. Briefly, primary neuron and microglia were plated at 2 × 10^6^ cells/well in 6-well plates 1 d before transfection. For each well, GPER-siRNA pool or an equal amount of NC- siRNA mixed with Lipofectamine 3000 was added to the cells. Cells were then cultured in the incubator at 37 °C and in 5% CO_2_. Forty-eight hours after the initial transfection, cells were analyzed by Western blotting to confirm the GPER knockdown or treated with β-alanine (10 ng/ml) for 24 h further, after which cytokine content was detected.

### Transcriptomics studies library preparation for transcriptome sequencing

DRG tissues of three rats in the sham, SNI, and SNI + G15 groups were randomly selected for transcriptome analysis. We extracted total RNA from approximately 150 mg of DRG tissue using a MirVana total RNA extraction kit (Ambion, Carlsbad, CA, USA). RNA levels were measured using the Qubit^®^ RNA Assay Kit and a Qubit^®^ 2.0 Fluorometer (Life Technologies, Carlsbad, CA, USA). We assessed RNA integrity using an RNA Nano 6000 Assay Kit of the Bioanalyzer 2100 system (Agilent Technologies, CA, USA). We used 1.5 μg of RNA per sample as the follow-up test material for RNA sample preparation. Whole transcriptome profiling was performed using NEBNext^®^ Ultra™ RNA Library Prep Kit for Illumina^®^ (NEB, Ipswich, MA, USA) following the manufacturer’s instructions [36].

#### Clustering, sequencing, quantification of gene expression and KEGG enrichment analysis of differentially expressed genes

We performed clustering of index-coded samples using a CBOT Cluster Generation system using HiSeq 4000 PE Cluster Kit (Illumina). Next, we performed RNA sequencing (150 bp, pair-ends) using standard Illumina HiSeq 4000 platform protocols. All downstream analyses were based on high-quality clean data. Gene FPKM was calculated by adding the FPKM of each genome transcript. The differential expression of both conditions was analysed using DESeq2R software package (1.26.0). For genes with FPKM values ≥ 1 in at least one sample, significant between-group differences in expression were determined according to the criteria as follows: *p* < 0.05 and |log2FoldChange|≥ 0.5. We used the Functional Annotation Bioinformatics Microarray Analysis (DAVID) database [37] to perform KEGG enrichment analysis of DEGs. DEG enrichment in KEGG pathways was examined using KEGG Orthology Based Annotation System v3.0 software. *p*-values were calculated using one-way ANOVA based on the normalized dataset. KEGG terms (*p* < 0.05) were considered significantly enriched by DEGs.

### Liquid chromatograph–mass spectrometer and metabolomics analysis

We randomly selected the DRG samples of 36 rats in the sham, SNI, and SNI + G15 groups for metabolomics analysis. Collect and store each tissue sample (~ 10 mg) in an Eppendorf Safelock microcentrifuge tube with 10 pre-chilled zirconia beads and 20 μl deionized water. The samples were homogenized for 3 min, and 120 μl of methanol was added to extract the metabolites containing the internal standard. Samples were homogenized for an additional 3 min and then centrifuged at 18,000*g* for 20 min. The supernatant was then transferred to a 96-well plate. The following procedures were performed on an Eppendorf epMotion workstation (Eppendorf Inc., Hamburg, Germany). 20 μl of the prepared derivatization reagent was freshly added to each well. The plate was sealed and derivatization was performed at 30 °C for 60 min. The derivatized samples were evaporated for 2 h. Add 330 μl of ice-cold 50% methanol solution to reconstitute the sample. Plates were then stored at − 20 °C for 20 min, and then centrifuged at 4000*g* for 30 min at 4 °C. Transfer 135 µl of supernatant to a new 96-well and add 10 µl of internal standard to each well. Serial dilutions of derived standards were added to the left wells. The plate was finally sealed for LC–MS analysis. A ultra-performance liquid chromatography coupled to tandem mass spectrometry (UPLCMS/MS) system (ACQUITY UPLC-Xevo TQ-S, Waters Corp., Milford, MA, USA) was used to quantitate all targeted metabolites in this project. The optimized instrument settings are briefly described below. The instrument performance optimization and routine maintenance were performed every week. (LC–MS) analysis an Agilent (Palo Alto, CA) HPLC 1200 system coupled with a 6490 MSD time‐of‐flight mass spectrometer (Agilent Corporation, Santa Clara, CA, USA) were utilized for LC‐MS analysis. The peak area of each metabolite was integrated using Mass Hunter Workstation Software Quantitative Analysis (Agilent) [38].

### Electrophysiological recordings

All recordings were performed on small- and medium-diameter (20–35 μm) neurons as previously described [39]. Coverslips with DRG neurons were mounted in a small flow-through chamber positioned on the stage of an inverted microscope (Nikon Eclipse Ti, Tokyo, Japan) to select DRG cells with smooth membrane surfaces and good translucency. We continuously perfused coverslips with gravity-driven bath solution. We performed standard whole-cell patch-clamp recordings of isolated DRG neurons at room temperature (22 °C) using an EPC-10 amplifier and the PULSE program (HKA Electronics, Lambrecht, Germany). Membrane capacitance was read from the amplifier using PULSE to measure the size of cells and current densities. Glass pipettes (3–5 MΩ) were prepared using a Sutter P-87 puller (Sutter Instruments, Novato, CA, USA). Action potentials were elicited by a series of depolarizing currents from 0 to 500 pA (150 ms) in 50-pA step increments under the current clamp mode to measure the current threshold (rheobase) in the vicinity of the explosive action potential current. The current was altered at 10-pA steps, i.e. the minimal current that evoked an action potential, which is a parameter for excitability. The recorded signal was amplified using a MultiClamp 700B amplifier (Molecular Devices, LLC, Sunnyvale, CA, USA), filtered at 10 kHz, and converted by an Axon Digidata 1550A D/A converter (Molecular Devices) at a 10–20 kHz sampling frequency. Voltage errors were minimized using 80–90% series resistance compensation; further, we used linear leak subtraction for all recordings. For the current clamp experiments, the bath solution contained the following (in mM): 140 NaCl, 5 KCl, 2CaCl_2_, 2MgCl_2_, 10 d-glucose, and 10 HEPES. The pH was adjusted to 7.4 using NaOH. The pipette solution contained the following (in mM): 30 KCl, 100 K-aspartate, 5 MgCl_2_, 2 Mg-ATP, 0.1 Na-GTP, and 40 HEPES, with adjustment of the pH to 7.2 using KOH. All chemicals were obtained from Sigma.

### Statistical analysis

**Fig. 1:**
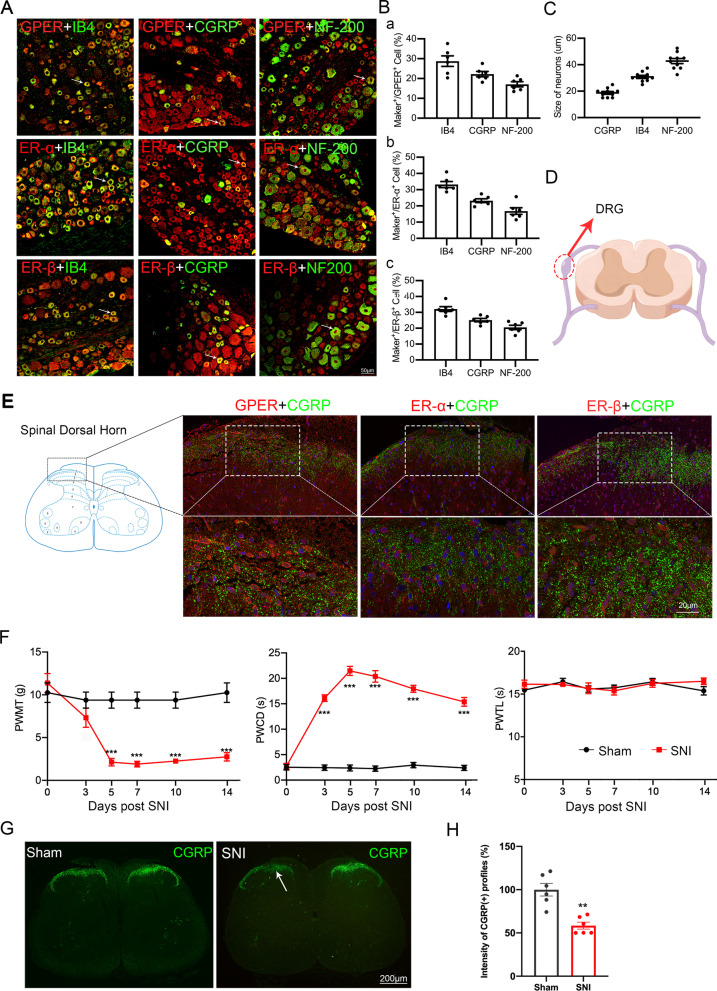
Distribution and expression of oestrogen receptors (ERs) in the dorsal root ganglion (DRG) and spinal dorsal cord (SDH), spared nerve injury (SNI) model builded. **A** Immunofluorescence double labelling revealed that ERs was colocalized with IB4 (a marker of nonpeptidergic C-type neurons), CGRP (a marker of peptidergic C-type neurons), and NF-200 (a marker of A-type neurons). White arrows refer to colabelled neurons, scale bar = 50 μm. **B** **a**–**c** The percentage of IB4-, CGRP-, and NF-200-positive (green) neurons relative to ERs (red) positive cells. **C** Neuronal diameter size of IB4-, CGRP-, and NF-200-positive neurons. **D** Diagram of the sampling site of DRG tissue. **E** Double staining showing the colocalization of oestrogen receptor-α, oestrogen receptor-β, and G protein-coupled oestrogen receptor (red) with the CGRP (green) in the rat SDH. The image in the white square is the zoomed-in image of the area below the corresponding image. **F** Mechanical allodynia and cold hyperalgesia developed in SNI rat paws compared to Sham rats from the third day after SNI until at least day 14, thermal hyperalgesia was not significant (*n* = 8), Mann–Whitney U test. ^***^p < 0.001, versus sham group. **G** Loss of CGRP(+) terminals in the SNI ipsilateral SDH (arrow), two-tailed unpaired Student’s t test. ^**^p < 0.01, versus sham. **H** Quantification of the number of CGRP(+) terminals in sham and SNI rats (*n* = 6 rats). Scale bar = 200 μm. *DRG* dorsal root ganglion, *SDH* spinal dorsal horn

All data are expressed as mean ± SEM of three independent experiments. Prior to further statistical analysis, the normal distribution hypothesis of the test data and homogeneity of variance were examined. Statistical analyses were performed using SPSS 20.0 (SPSS Inc., Chicago, IL, USA) and Graphpad 9.0 (California, USA). Figures 1D and 8 were drawn By Figdraw (www.figdraw.com). We analysed PWMT, PWCD, and PWTL using repeated-measures analysis of variance. Multiple between-group comparisons at each time point were conducted using Kruskal–Wallis test followed by Dunn’s multiple comparison test. Among-group comparisons of the western blot, PCR, and patch-clamp data were conducted using one-way analysis of variance followed by Tukey’s post hoc tests. Between-group comparisons were performed using Student’s *t*-test. Statistical significance was set at *p* < 0.05.

## Results

### Three ERs were expressed in the pain-related area of DRG small and medium-sized neurons and the SDH

To determine the function of ERs in the pain-related area of DRG neurons and SDH, we initially used immunofluorescence double staining to characterize the expression of ERs in DRG neurons. Immunofluorescent double staining experiments showed that ERs colocalized with IB4, CGRP, and NF-200 markers. The percentages of IB4- (labelled C-type nonpeptidergic neurons), CGRP- (labelled C-type peptidergic neurons), and NF-200- (labelled A-type neurons) positive neurons relative to the percentage of ERs -positive cells were assessed (*n * = 6 in each group; Fig. 1B). GPER colocalized with IB4-labelled neurons (28.75 ± 2.635%), CGRP-labelled neurons 22.25 ± 1.345%, NF-200-labelled neurons (17.1 ± 1.29%) (Fig. 1Ba); IB4-labelled neurons (33.16 ± 1.825%), CGRP-labelled neurons (23.19 ± 1.166%), NF-200-labelled neurons (16.82 ± 2.05%) (Fig. 1Bb); ERβ colocalized with IB4-labelled neurons (32.1 ± 1.521%), CGRP-labelled neurons (25.16 ± 1.043%), NF-200-labelled neurons (20.52 ± 1.38%) (Fig. 1Bc). These results showed that ERs were mainly located in A- and C-type neurons in the DRG. The neuronal diameter size ranges of IB4, CGRP, and NF-200 were 30.8 ± 1.167, 18.75 ± 1.07, and 42.75 ± 1.917, respectively (*n * = 10 in each group) (Fig. 1C). Oestrogen receptors expression, mainly in medium/small-sized but also in large DRG neurons. After obtaining DRG tissues (Fig. 1D), the pain-related area in the dorsal horn of the spinal cord was labelled with CGRP, and we found that three oestrogen receptors are expressed in this area (Fig. 1E). Expression of ERs in the pain-related cells and area indicated that oestrogen receptors may be involved in the regulation of superficial sensations such as pain.

### The development of cold and mechanical allodynia and CGRP loss in the SDH successfully established a neuropathic pain model of SNI in SD rats

Paw withdrawal mechanical threshold in the sham and SNI groups to von Frey hair stimuli 1 day before and 3, 5, 7, 14, and 21 days after the surgery were generated. Mechanical stimuli were applied to the left hind paw of rats with von Frey filaments, and these data were compared with the sham group at the same time point. Acetone (100 μl) was used on the left hind paw of the rats, to measure time courses of the withdrawal duration to cold stimuli in the sham and SNI groups. Cold and mechanical hyperalgesia began to appear on the third day and peaked on the fifth day and persisted to at least 14 days (Fig. 1E, *n * = 8). Time courses of the withdrawal latency to thermal stimuli were determined in the sham and SNI groups. Radiant heat stimulus (IR 50) was applied to the left paws of the rats. There was no significant change in thermal stimulation. In the SNI group, significant CGRP loss was noted on the SNI side on postsurgery day 5 (Fig. 1F). As shown in Fig. 1G, compared to the sham group (47.12 ± 7.283), the SNI group (21.18 ± 3.98) fluorescence intensity decreased, (*p* < 0.01, two-tailed unpaired Student’s t test). Therefore, we chose the fifth day after SNI for the sampling and drug intervention time points [10].

### GPER and ERα were significantly increased in the DRG but not the SDH, and cold and mechanical hyperalgesia were induced by intrathecal injection the GPER-specific agonist G1 in normal rats

**Fig. 2:**
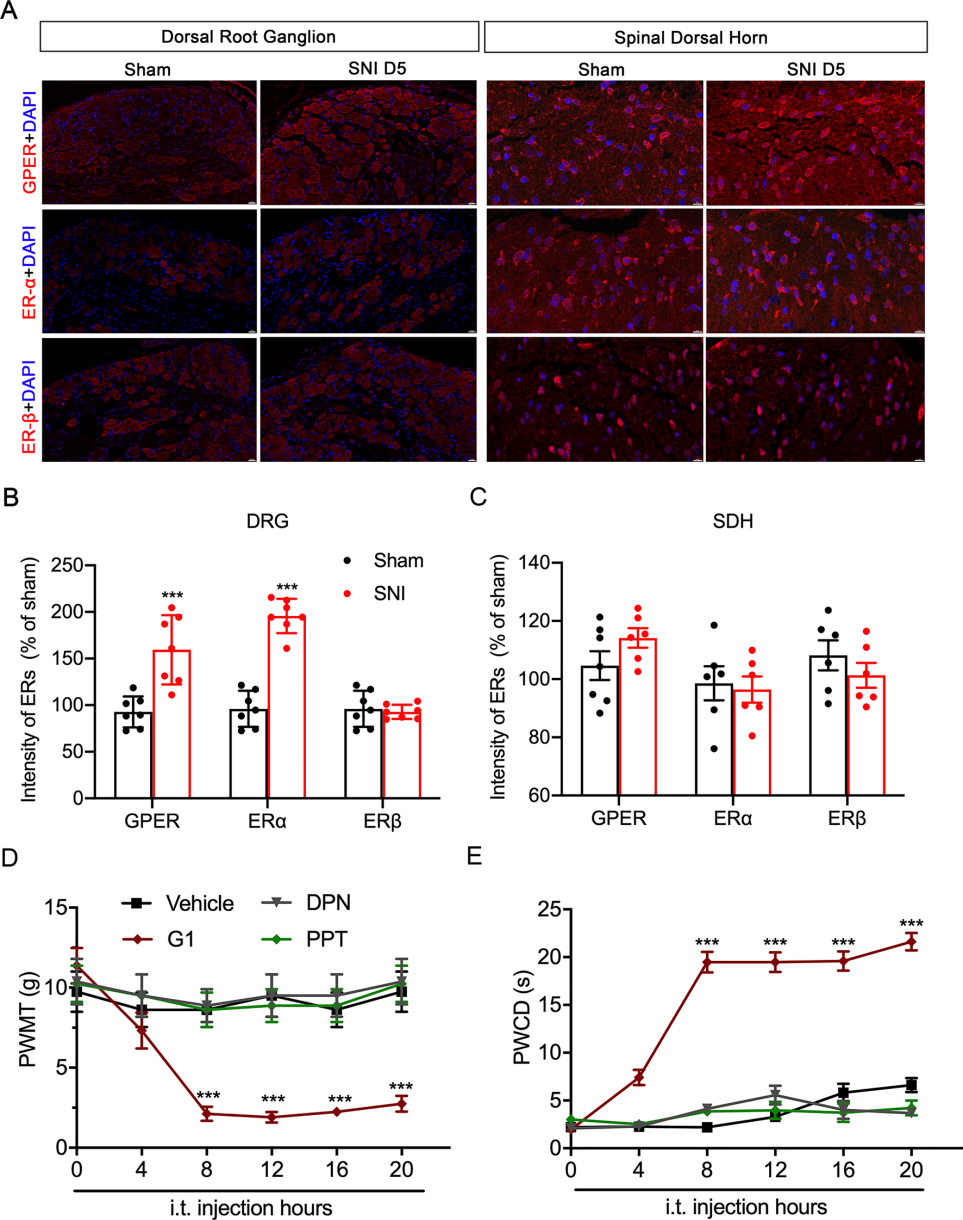
GPER not ERα or ERβ, contributes to spared nerve injury (SNI)-induced pain behaviours in the dorsal root ganglion (DRG) but not in the spinal dorsal horn (SDH). **A** Quantification of fluorescence intensity showed the G protein-coupled oestrogen receptor, oestrogen receptor-α and oestrogen receptor-β expression in DRG and dorsal spinal cord of SNI rats compared to sham rats (*n* = 6–7 sections from three rats). Two-tailed unpaired Student’s t test.^***^p < 0.001, ^**^p < 0.01 versus Sham. Scale bars = 20 μm (DRG) and 10 μm (SDH). **D**, **E** Intrathecal injection of the G protein-coupled oestrogen receptor-1 agonist G1 (0.2 μg/μl, 1 μl), the oestrogen receptor-α agonist PPT (0.02 ng/μl, 1 μl) and the oestrogen receptor-β agonist DPN (0.02 ng/μl, 1 μl), directly into the subarachnoid space induced mechanical allodynia and cold hyperalgesia. Kruskal–Wallis test followed by Dunn’s multiple comparison test. ^***^p < 0.001 versus vehicle (10% dimethyl sulfoxide); *n* = 8 for each group

Immunofluorescence staining (Fig. 2A) detection of rat ipsilateral L4-6 DRGs at the spared nerve injury (SNI) site showed that the fluorescence intensity of GPER and ERα were higher than those in sham-operated rats on day 5 after the operation (GPER, 159.4 ± 14.02% of SNI group and 92.76 ± 6.296% of Sham group, *p* < 0.001; ERα, 195.7 ± 7.298% of SNI group and 96.08 ± 6.962% of Sham group, *p* < 0.001 two-tailed unpaired Student’s t test, Fig. 2B, C). Notably, the ERs protein fluorescence intensity and mRNA levels in SDH had no change (Additional file 1: Fig. S1). The DRG is the primary sensory neuron for pain [40], so we hypothesize that the increased receptors in the DRG may be involved in the occurrence of pain. Therefore, normal rats were administered with G1, PPT, and DPN, which are GPER-specific, ERα-specific, and ERβ-specific agonists, respectively. Only GPER-specific agonists could induce hyperalgesia similar to the SNI model, the effect of G1 lasted from the 8th hour of administration to at least 20 h (Fig. 2D, E). After intrathecal injection of the agonist in normal rats, the GPER-specific agonist G1 developed mechanical and cold hyperalgesia from the 8th hour of injection and continued to 20 h (PWMT, from 0 h 11.375 ± 1.101 g to 8th hour 2.125 ± 0.441 g, to 20th hour 2.75 ± 0.491 g, *p* < 0.001; PWCD, from 0 h 1.966 ± 0.404 s to 8th hour 19.48 ± 1.075 s, to 20th hour 21.611 ± 0.927 s, *p* < 0.001, Kruskal–Wallis test followed by Dunn’s multiple comparison test).

### G15 attenuated cold and mechanical allodynia in SNI rats, and GPER is involved in neuropathic pain by regulating α2-GABAA

**Fig. 3:**
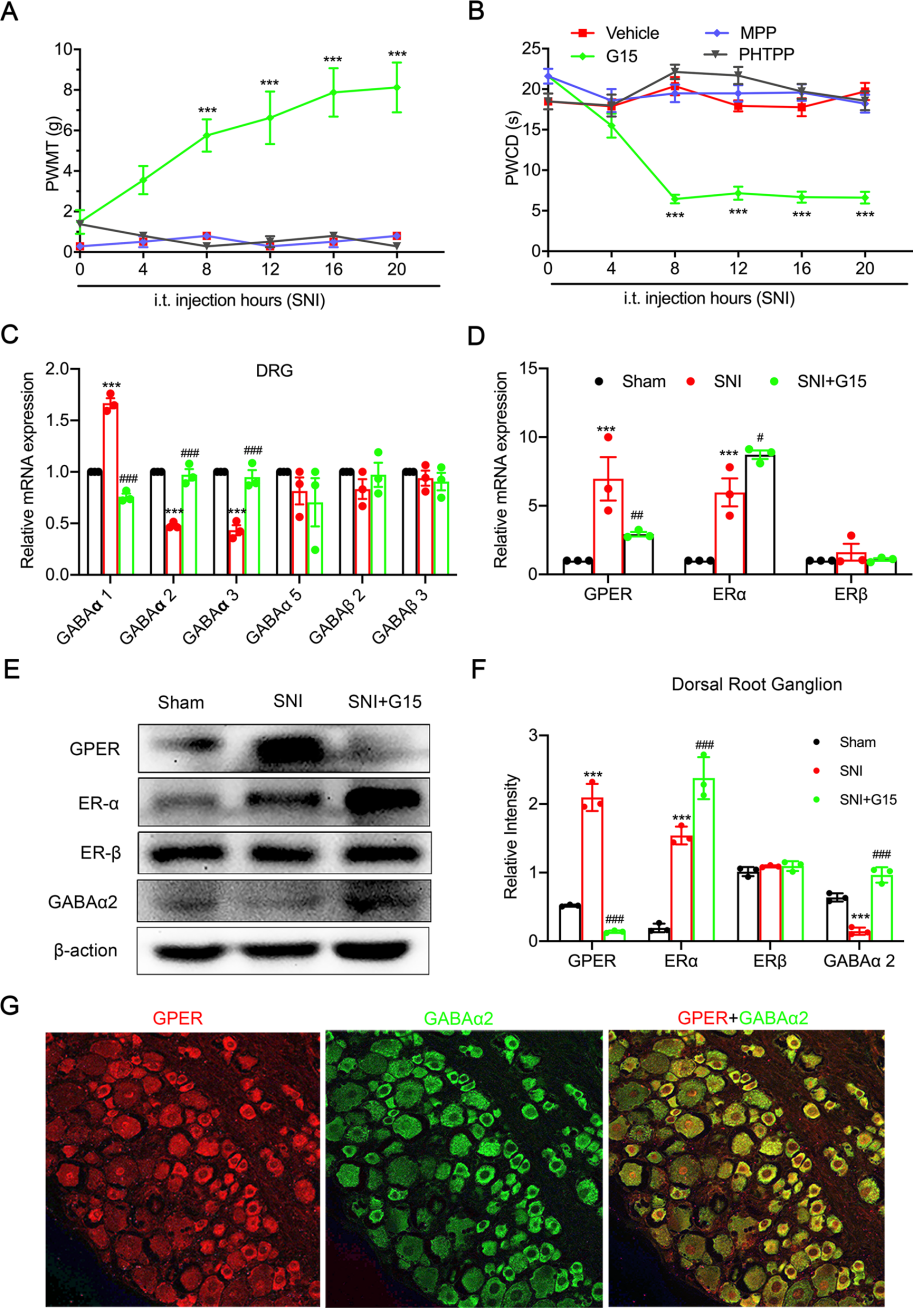
Inhibiting GPER expression in the DRG and SDH alleviates spared nerve injury (SNI)-induced pain behaviours in rats and simultaneously restores GABAα2 expression. **A**, **B** The oestrogen receptor-α antagonist (MPP; 1 ng/μl, 1 μl) or the G protein-coupled oestrogen receptor antagonist (G15; 1.8 μg/μl, 1 μl) oestrogen receptor-β antagonist (PHTPP; 1 ng/μl, 1 μl) was intrathecally injected into the subarachnoid space of SNI rats on Day 5 to detect mechanical allodynia and cold hyperalgesia. ^***^p < 0.001 versus vehicle (10% dimethyl sulfoxide; Kruskal–Wallis test followed by Dunn’s multiple comparison test). **C** Quantification of the mRNA content of GABA receptor subunits in the DRG defined. Error bars in all panels represent S.E.M. (*n* = 3, one-way ANOVA. ^***^p < 0.001 versus Sham; ^###^*p* < 0.001 versus SNI). **D** Intrathecal injection of the G protein-coupled oestrogen receptor-1 antagonist (G15; 1.8 μg/μl, 1 μl) into the subarachnoid space of SNI rats on day5 to detect mRNA content of oestrogen receptor-α, oestrogen receptor-β, and G protein-coupled oestrogen receptor in the DRG (*n* = 3, one-way ANOVA. ^***^p < 0.01 versus Sham; ^#^*p* < 0.05, ^##^*p* < 0.01 versus SNI). **E**, **F** Western blot images and quantification for GPER, ERα, ERβ, and GABAα2 in the DRG after sham, SNI and intrathecal injection of G15 of rats. (*n* = 3, one-way ANOVA. ^***^p < 0.001 versus Sham; ^###^*p* < 0.001 versus SNI). **G** Immunofluorescence coexpression of GPER and GABAα2 in the dorsal root ganglia of rats on Day 5 after SNI. Scale bars = 50 μm, *n* = 6

To evaluate the potential function of GPER in neuropathic pain, we intrathecal injection the GPER-specific blockers G15 on day 5 of SNI. In all SNI + G15 group rats that received G protein-coupled oestrogen receptor-1 antagonist (G15; 1.8 μg/μl, 1 μl), cold and mechanical allodynia induced by SNI was partially reversed, and the effect persisted from the 8th hour and continued to 20 h of behavioural testing (PWMT, from 0 h 1.483 ± 0.591 g to 8th hour 5.75 ± 0.796 g, to 20th hour 8.125 ± 1.231 g, *p* < 0.001; PWCD, from 0 h 21.611 ± 0.927 s to 8th hour 6.453 ± 0.546 s, to 20th hour 6.620 ± 0.728 s, *p* < 0.001, Kruskal–Wallis test followed by Dunn’s multiple comparison test). Oestrogen receptor-α antagonist (MPP; 1 ng/μl, 1 μl) and oestrogen receptor-β antagonist (PHTPP; 1 ng/μl, 1 μl) did not affect PWMT and PWCD (Fig. 3A, B). In the DRG, α2-GABAA plays an important role in the pathophysiology of NPP caused by sciatic nerve injury [41]. By detecting the mRNA expression of GABAA subunits (Fig. 3C), the mRNA levels were consistent with the western blotting results, indicating that GPER is involved in neuropathic pain by regulating α2-GABAA (Fig. 3F, *p* < 0.001, one-way ANOVA followed by Tukey’s test). GPER and ERα protein and mRNA levels of were both upregulated post-SNI operation, and SNI-induced loss of GABAα2 restored after G15 in the sheath (Fig. 3D–F). These results indicated that GPER may be involved in the regulation of superficial sensations such as pain, through α2-GABAA. Double immunofluorescence staining showed that GPER and α2-GABAA are colocalized in the dorsal root ganglia neurons (Fig. 3G). α2-GABAA has been shown to play an important role in neuropathic pain, further indicating that GPER is involved in the regulation of pain.

### Transcriptomics testing found that GPER was involved in neuropathic pain regulation through metabolic pathways while regulating neuroinflammation

**Fig. 4:**
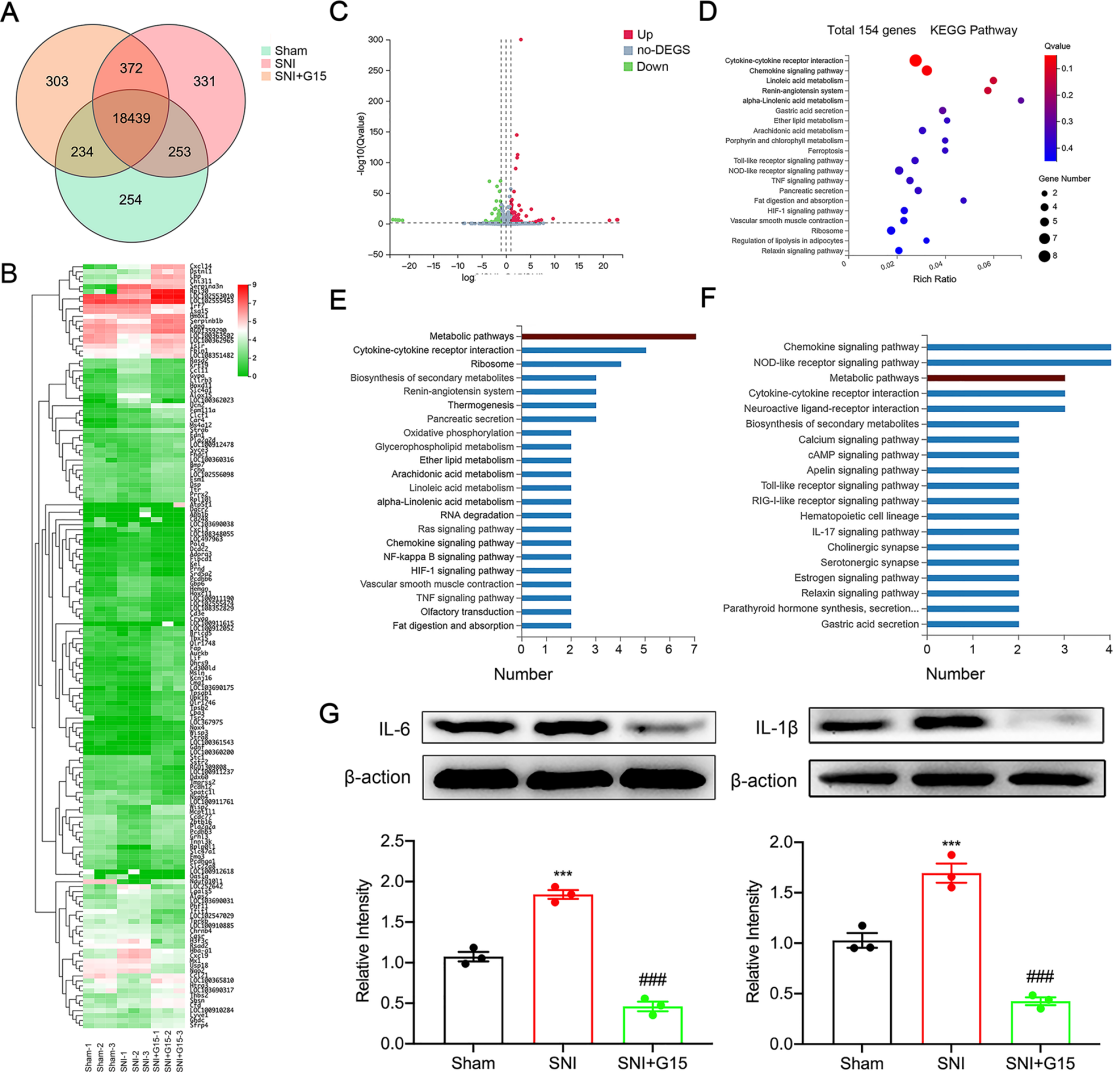
RNA-Seq identifies mRNA expression changes in the dorsal root ganglion (DRG) of spared nerve injury (SNI) rat models and G15-treated rats, and inflammation occurs synchronously. **A** Venn diagram showing the overlapping of DEGs in DRG from Sham, SNI, SNI + G15 rats. **B** Heatmap illustrations of hierarchical clustering analysis of differentially expressed mRNAs (DEmRNAs) in DRG from sham, SNI, SNI + G15 group rats. **C** Volcano plots of DEmRNAs in the DRG of SNI + G15 group rats vs. SNI rats. Red points refer to upregulated DEGs and green points refer to downregulated DEGs, and grey spots indicate non-DEGs. **D** Bubble plots showing the top 20 significant pathways for upregulated DEmRNAs. Downregulated DEmRNAs. **E**, **F** KEGG pathway analysis of DEmRNAs. E, The top 22 significant biological processes, molecular functions, and cellular components of upregulated DEmRNAs. F, The top 19 significant biological processes, molecular functions, and cellular components of downregulated DEmRNAs. The dotted line indicates a *p* value < 0.001. **G** Protein expression levels of IL-1β and IL-6 in DRG from Sham, SNI, SNI + G15 rats. (*n* = 3, one-way ANOVA, ^**^p < 0.01 versus sham; ^##^*p* < 0.01 versus SNI)

We examined the gene expression profiles with a focus on mRNA in neurons of sham, SNI, and SNI + G15 rats using RNA-Seq. A total of 18,439 genes were expressed in the three groups (Fig. 4A). A total of 154 genes exhibited differences between groups. Compared with the SNI + G15 group, 90 genes were significantly upregulated in the SNI group, and 64 genes were significantly downregulated (Fig. 4B). KEGG analysis used to 154 total genes enrich (Fig. 4C, D), while KEGG analysis of upregulated and downregulated genes showed that the metabolic pathway exhibited the most significant difference(Fig. 4E, F). Based on the date, we can perform metabolomics testing on the three groups to identify the key metabolites that cause GPER changes. Unexpectedly, we discovered that GPER regulate the expression of the inflammatory factors IL-6 and IL-1β, and that GPER blockers can significantly reduce neuroinflammation (Fig. 4G).

### Metabolomics tests showed that β-alanine induces GPER upregulation

**Fig. 5:**
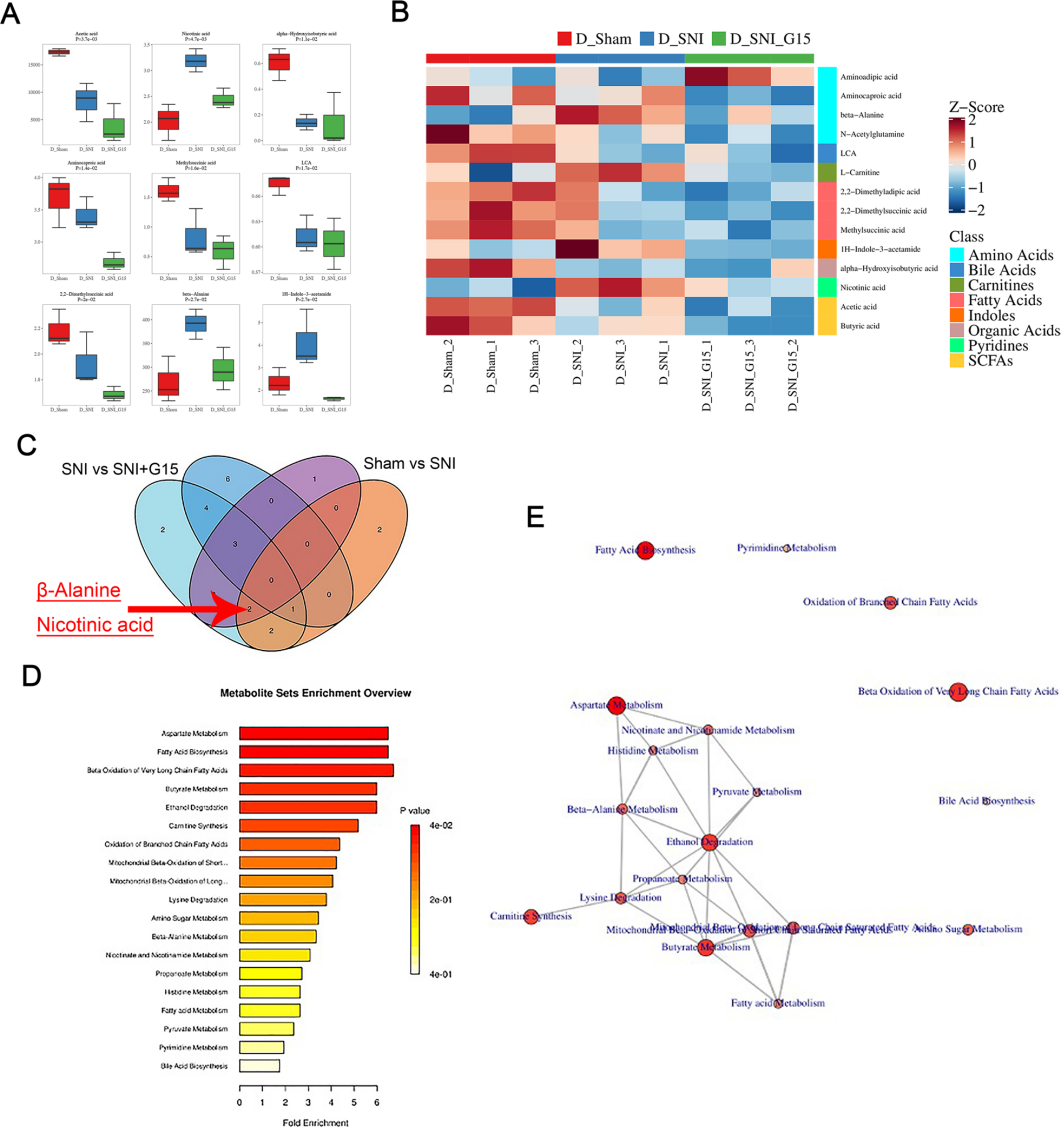
Metabolomics reveals that β-alanine metabolism is involved in post-spared nerve injury (SNI) GPER upregulation. **A** Box charts of the relative trends of top nine metabolites involved in the important metabolic pathways that were identified. Data are expressed as the mean ± SEM. **B** Heatmap of metabolites in DRG of rats in the sham, SNI, and SNI + G15 groups. Red indicates higher levels and blue indicates lower levels. **C** Venn diagram showing the overlapping of metabolites in DRG from Sham, SNI, and SNI + G15 rats based on pairwise comparison. The number represents the number of metabolites that change together in a pairwise comparisons. **D** Metabolite sets enrichment overview analysis. **E** PPI network analysis of metabolites involved in GPER response-related pathways in the DRG of SNI model rats. Metabolites related to pain response-related pathways identified by GO and KEGG analyses were subjected to PPI network analysis. A larger circle reflects more interactions and vice versa

According to the results of genomics, targeted metabolomics detected greater than 300 metabolites and found that 14 substances exhibit differences between groups: β-alanine; aminocaproic acid; 1H-Indole-3-acetamide; nicotinic acid; aminoadipic acid; N-acetylglutamine; acetic acid; alpha-hydroxyisobutyric acid; methylsuccinic acid; lithocholic acid; L-carnitine; 2,2-dimethyladipic acid; 2,2-dimethylsuccinic acid; and butyric acid (Fig. 5A, B). The sham group compared with the SNI group, and the SNI group was compared with the SNI + G15 group. The substances exhibiting alterations included β-alanine and nicotinic acid (Fig. 5C). We were especially interested in pathways involved in pain and inflammatory responses given that these pathways may be involved in mediating neuroinflammation and pain mechanisms in SNI model rats. Therefore, we continued to perform PPI analysis of the metabolites that are related to the processes we identified from KEGG analysis, and β-alanine may represent a key factor causing the increase in GPER (Fig. 5D, E).

### β-Alanine and GPER positively interact and induce DRG neuron excitability and neuroinflammation similar to that noted in SNI-induced neuroinflammation

**Fig. 6:**
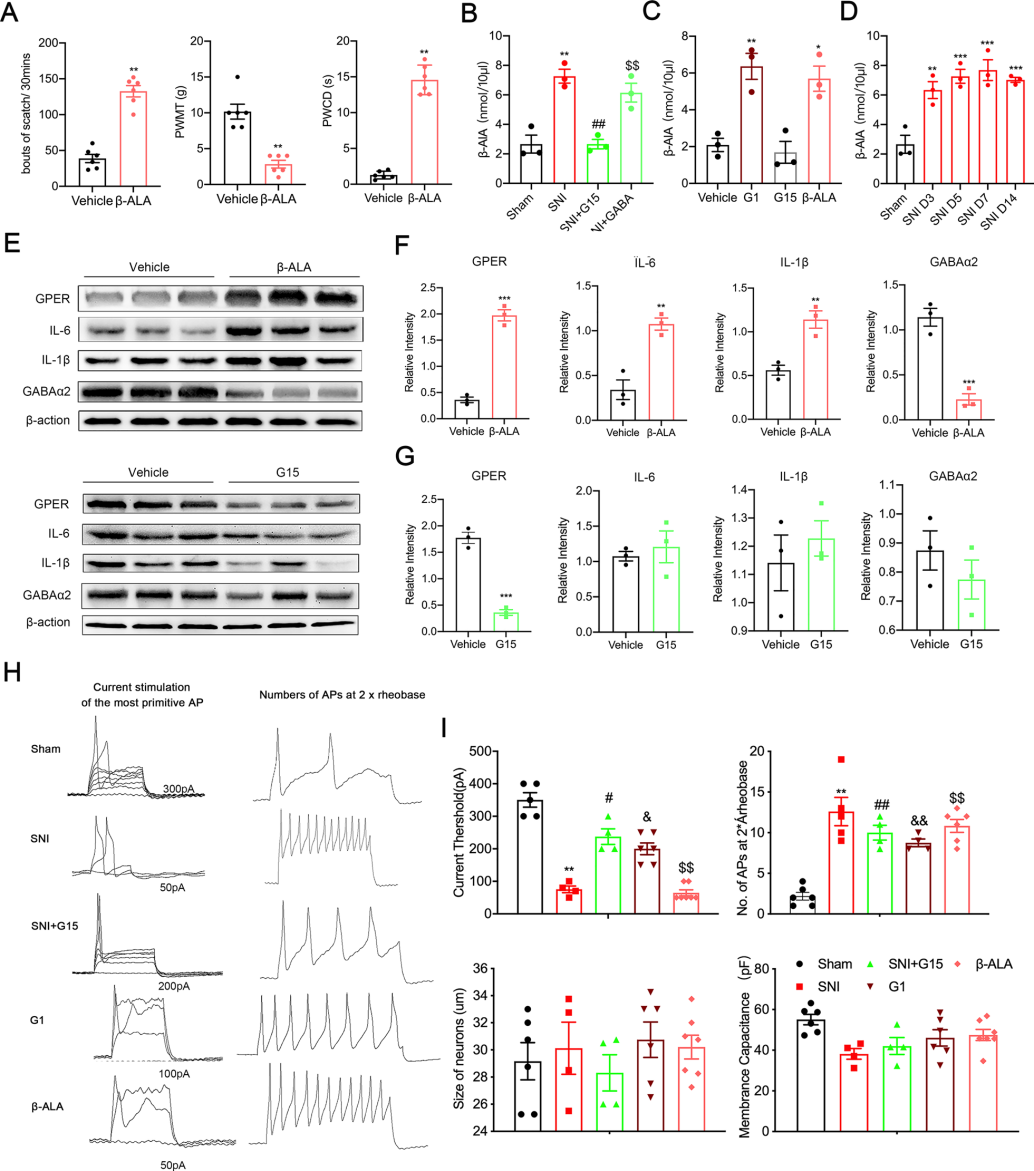
GPER/β-alanine positive feedback interaction in the dorsal root ganglion (DRG) mediating neuronal sensitization and neuroinflammation responses. **A** Itch allodynia and mechanical allodynia and cold hyperalgesia were developed rats administered intrathecal β-alanine rat after 24 h (*n* = 6, two-tailed Student’s t test. ^**^p < 0.01 versus Vehicle). **B** β-Alanine content in the DRG of rats in the Sham, SNI, and SNI + G15, and SNI + GABA groups (*n* = 3, one-way ANOVA. ^**^p < 0.01 versus sham; ^##^*p* < 0.01 versus SNI; ^$$^*p* < 0.01 versus sham). **C** β-Alanine content in the DRG of rats in the Vehicle, G1, and G15 groups. two-tailed Student’s t test. ^*^p < 0.05, ^**^p < 0.01 versus Vehicle. **D** β-alanine content in DRG of rats in the Sham and SNI Day 3, 5, 7, and 14 groups. (One-way ANOVA followed by Tukey’s test. ^**^p < 0.01, ^***^p < 0.001 versus the sham group.); *n* = 3. **E** Western blot images for GPER, ERα, ERβ, IL-6, IL-1β, and GABAα2 in the DRG after intrathecal injection of β-alanine and G15 in rats. **F** GPER, IL-6, and IL-1β protein level were increased, GABAα2 downregulated in the dorsal root ganglion (DRG) of β-alanine group rats. (*n* = 3, two-tailed Student’s t test. ^**^p < 0.01, ^***^p < 0.001 versus vehicle). **G** Quantification for GPER, ERα, ERβ, and GABAα2 in the DRG after intrathecal injection of G15. Except GPER protein downregulated, IL-6, IL-1β, and GABAα2 had no significant. **H** Current threshold (rheobase) was determined as the current required for activating the first action potential. On the left, representative traces of action potentials (APs) evoked by current injections into DRG neurons from Sham, SNI, SNI + G15, G1, and β-alanine groups; *n* = 4–6 neurons per group; on the right, the number of action potentials produced at the corresponding 2 × rheobase. **I** Statistical analysis revealed the rheobase; size of neurons, membrane capacitance, and number of action potentials (APs) at 2 × rheobase in DRG neurons. *n* = 4–6 neurons per group. One-way ANOVA followed by Tukey’s test. ^**^p < 0.01 versus sham; ^#^*p* < 0.05, ^##^*p* < 0.01 versus SNI; ^&^*p* < 0.05, ^&&^*p* < 0.01 versus sham; ^$$^*p* < 0.01 versus sham. β-ALA: β-alanine

To further determine the contribution of β-alanine to the increased GPER level noted in neuropathic pain, 10 μl of 10 mM β-alanine or 1% DMSO as vehicle was administered intrathecally to normal rats for 24 h for mechanical and cold hyperalgesia, and itching was significantly altered in rats (Fig. 6A, bouts of scatch/ 30 min, compared to the sham group 38.83 ± 5.816, the SNI group 132.7 ± 7.978 increased, *p* < 0.001; PWMT, compared to the sham group 10.172 ± 1.329 g, the SNI group 2.883 ± 0.542 g mechanical hyperalgesia, *p* < 0.01; PWCD, compared to the sham group 1.285 ± 0.546 s, the SNI group 14.57 ± 0.841 s increased, *p* < 0.001, two-tailed unpaired Mann–Whitney test). After administration of GPER blocker, the content of β-alanine was significantly lower than that after SNI. β-Alanine levels were not reduced after administration of GABA, so it can be inferred that β-alanine is upstream of GABA (Fig. 6B). We hypothesized that an interaction occurs between GPER and β-alanine. In normal rats, the β-alanine content was significantly increased after administration of the GPER agonist G1 (Fig. 6C). β-Alanine content increased after SNI, and the β-alanine content continued to accumulate until 14 days later (Fig. 6D). After the administration of β-alanine, western blotting was used to detect a significant increase in GPER protein levels, confirming a positive interaction between GPER and β-alanine and a cascade effect after SNI (Fig. 6E). Increased GPER levels and accumulation of β-alanine simultaneously upregulated downstream IL-6 and IL-1β levels and downregulated GABA levels to cause hyperalgesia and neuroinflammation (Fig. 6F). Normal rats administered GPER blocker G15 had no obvious effect on neuronal excitability and neuroinflammation, suggesting that GPER plays a role in pathological conditions (Fig. 6G). From a functional point of view, the patch-clamp technique verified that the increase in GPER and β-alanine caused an enhancement of DRG neuron excitability similar to that induced by SNI (Fig. 6H, I) and revealed the pathogenesis of neuropathic chronic pain.

### β-Alanine positively binds with GPER to excite neurons and participate in neuroinflammation with microglia

**Fig. 7:**
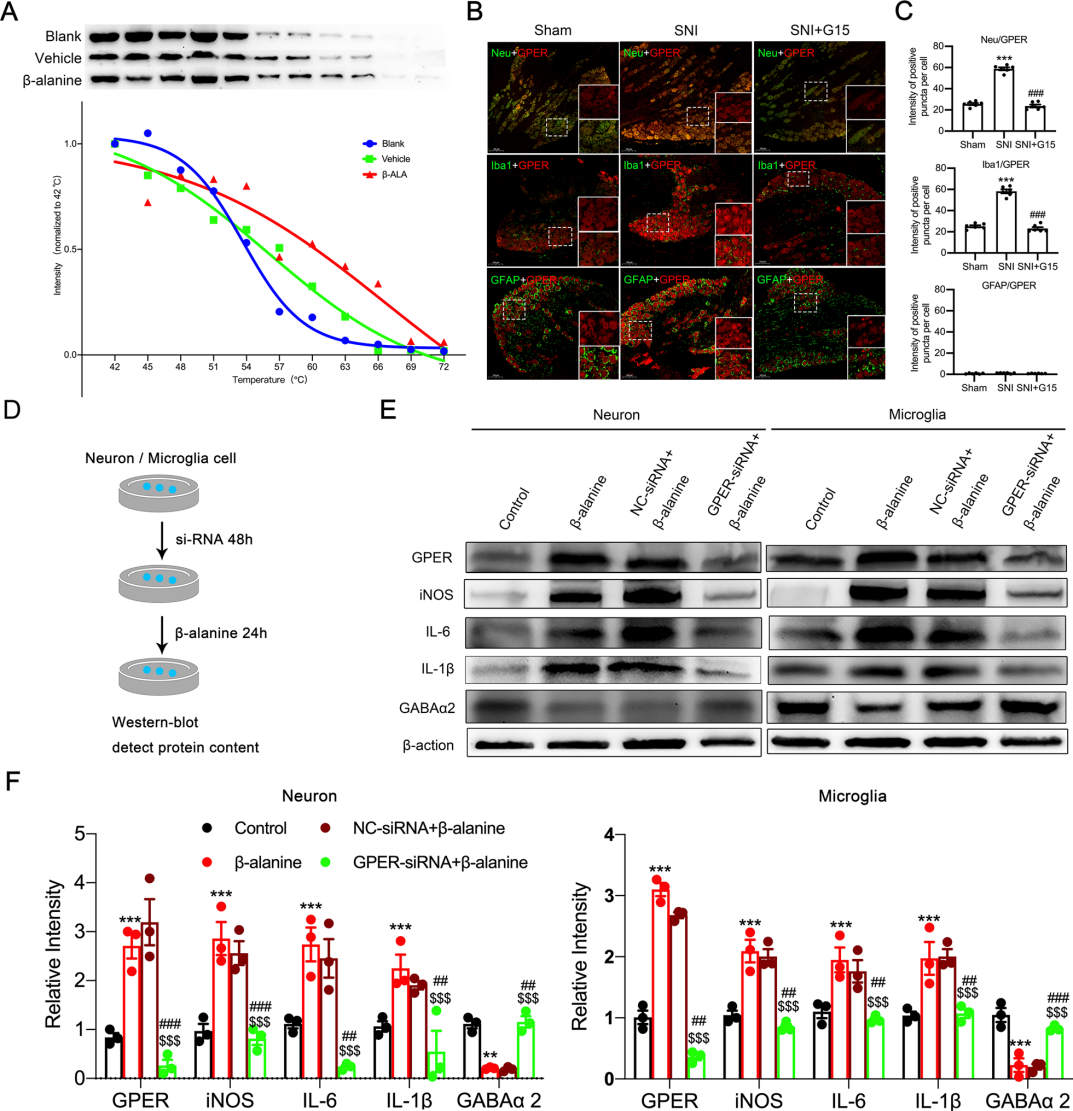
β-alanine binding to GPER in dorsal root ganglion (DRG) neurons and microglia cells in vitro. **A** Representative immunoblotting analysis of the protein lysates of DRG neurons isolated from blank control, Vehicle, and β-alanine rats DRG tissues and heated at a series of temperatures from 42 to 72 °C for 3 min. Statistical regression (4 parameter logistic regressions, *p* < 0.05) of separate immunoblots generating the cellular thermal shift assay curves for the enhanced thermal stability of GPER by β-alanine. (B and C) Representative images (**B**) and quantifications (**C**, *n* = 6 sections from three rat DRGs) showed the GPER protein coexpressed in three different types of cell neuron, microglia and astrocytes. The corner image in the white square is the zoomed-in image of the area in the smaller white square. **D** The protocol of neuron, and microglial cell transfection with GPER-siRNA and β-alanine incubation. **E** Western blot images for GPER, iNOS, IL-1β, IL-6, and GABAα2 in the neurons and microglia. **F** Western blotting analysis diminished the protein expression of GPER, iNOS, IL-1β, IL-6, and GABAα2 (*n* = 3). Two-tailed unpaired Student’s t-test. ^***^p < 0.001 versus control group; ^##^*p* < 0.01 ^###^*p* < 0.001 versus NC-siRNA + β-alanine group; ^$$$^*p* < 0.001 versus β-alanine

**Fig. 8:**
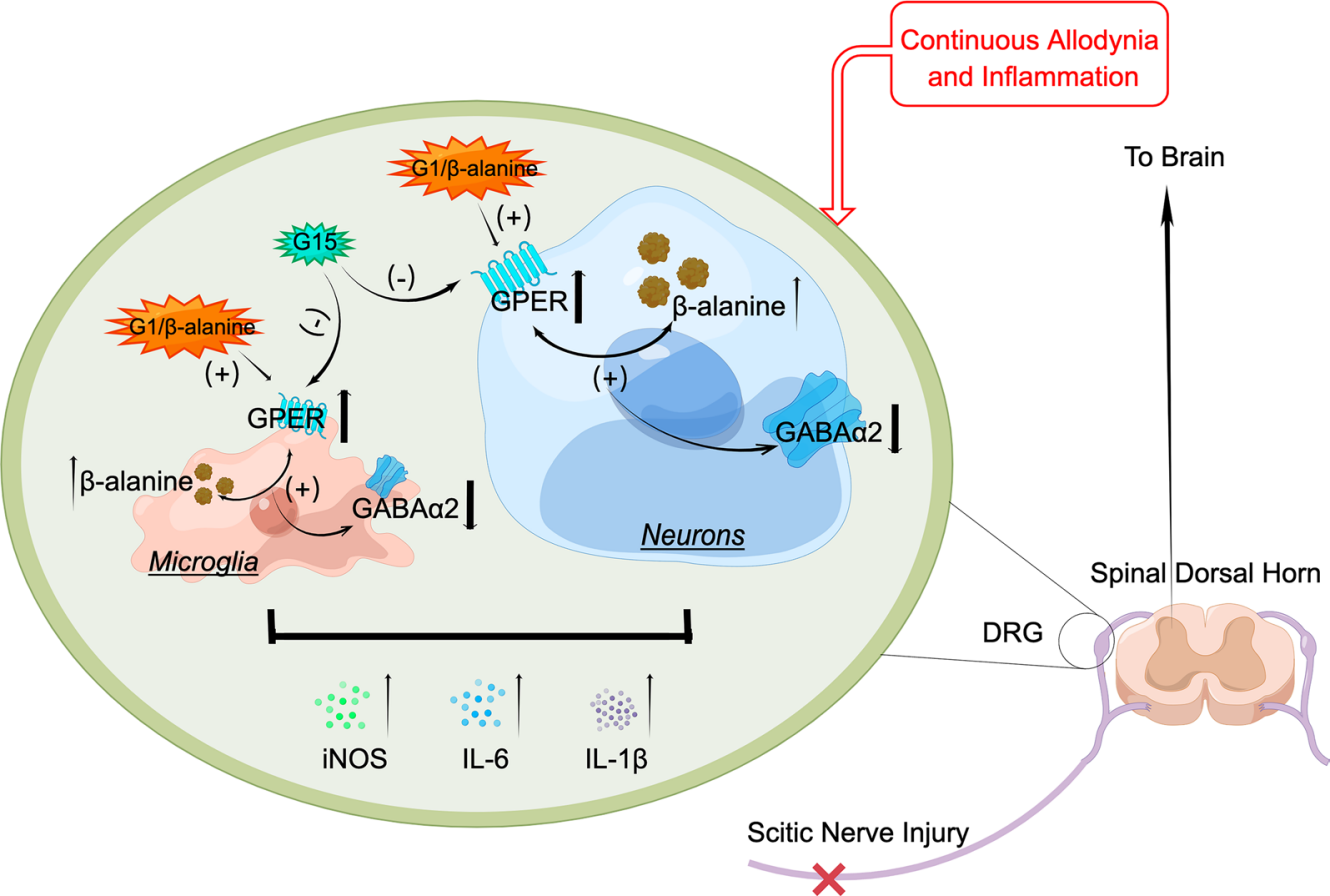
Mechanistic diagram of GPER/β-alanine mediating neuronal sensitization and neuroinflammation in modulating neuropathic pain. Schematic illustration demonstrates that the upregulation of GPER in neurons and microglial cells in the DRG signals to sensory neurons upon peripheral nerve damage, inducing an increase neuronal β-alanine while positively promoting GPER. Their combined effect further activates iNOS, IL-1β and IL-6 and regulates GABAα2. The positive GPER/β-alanine interaction in the DRG uncovers enhances pain sensation and promotes chronic pain development

Temperature gradient experiments showed that in DRG tissues treated with β-alanine, the of incubation shifts to the right, indicating that GPER can bind to β-alanine, thereby enhancing the stability of GPER and degrading it more slowly. Indicating that β-alanine can combine with GPER (Fig. 7A). Double immunofluorescence staining showed that GPER colocalized with neurons and microglia but not with astrocytes (Fig. 7B). GPER was downregulated in the SNI + G15 group compared with the SNI group (Fig. 7C). To pinpoint the location of action, we extracted the primary neurons and microglia of the DRG, and stimulation with exogenous β-alanine induced similar in vivo (Fig. 7D) changes of iNOS, IL-1β, IL-6, and GABAα2 proteins level. GPER protein expression levels were decreased after transfection with GPER-siRNA, but the above changes did not appear upon stimulation with exogenous β-alanine (Fig. 7E, F). GPER activation in the neurons and microglia of the DRG causes a positive association between β-alanine and iNOS, IL-1β and IL-6 activation and represses GABAα2 expression involved in post-SNI neuropathic pain and neuroinflammation development (Fig. 8).

## Discussion

Currently, neuropathic pain cannot be effectively treated given that its exact cause remains unclear [42, 43]. Sensitization is crucial for neuronal hyperactivity and is characterized by overreaction to normal stimuli [44]. Ectopic discharge is the electrophysiological basis of allodynia; moreover, the change of ion channels is the main reason for abnormal discharges and α2/α3 GABAA-mediated analgesia in neuropathic pain [10, 45]. An increasing number of studies have shown that oestrogen can affect pain by regulating ion channels; however, there are conflicting opinions regarding pain and analgesia [45]. Oestrogen mainly acts through its three receptors GPER, ERα, and ERβ [5]. However, few studies have investigated the role of ER in peripheral pain transduction. We observed significant post-SNI GPER upregulation; moreover, blocking GPER can relieved hyperalgesia. These findings demonstrated the possible mechanism of neuropathic pain onset and persistence from the perspective of material metabolism.

Oestradiol modulates the efficacy of synaptic inhibition by decreasing the dwell time of GABAA receptors at inhibitory synapses [23]. Mouse models of inflammatory and neuropathic pain have revealed that the novel α2-GABAA receptor is antihyperalgesic [17]. Therefore, we chose other receptors as the reference molecular biological indicators of pain. In this study, after establishing the SNI model, there were behavioural changes in mechanical and cold hyperalgesia were observed; moreover, no significant change in thermal pain was noted. In the follow-up study, we chose PWMT and PWCD as the behavioural reference indicators. On the SNI injury side, there was significant CGRP loss in the SDH as well as coexpression of the three ERs were noted. Moreover, our findings demonstrated that GPER, ERα, and ERβ are mainly expressed in pain-related small and medium-sized neurons. DRG neurons of various sizes have different responses to various stimuli [46]. Small- and medium-sized neurons specifically respond to harmful cold, thermal, chemical, and mechanical stimuli; therefore, they are considered multimodal nociceptors. Furthermore, the DRG and SDH are closely associated with pain transmission; moreover, we found that the three ERs are closely related to pain. Oestrogen could influence P2X3 expression via the ERα and GPER ERα to affect neuropathic pain, which may be mediated through the ERK pathway [47]. The deletion of the ER subunit α (ERα) in TRPV1 nociceptors abolishes IL-23- and IL-17-induced pain in females [48]. ER-β and G protein-coupled ER-1, but not ER-α, in the rostral anterior cingulate cortex are involved in pain-related aversion by modulating *N*-methyl-d-aspartate receptor-mediated excitatory synaptic transmission [49]. Behavioural tests revealed that the PWMT and PWCD peaked on the fifth day and lasted > 14 days in SNI animals. Therefore, we choose the fifth day as the timepoint for selecting animals for the experiment and drug intervention. SNI animals have been reported to maintain strong mechanical allodynia throughout an 85-day observation period [50].

We used immunofluorescence to detect the expression of three ERs in DRG neurons and SDH areas with CGRP deletion. On the fifth day of SNI, GPER and ERα expression were significantly increased in the DRG but not the SDH. Western blot and PCR results were consistent with those of immunofluorescence. G15, which is a specific blocker of GPER, can relieve hyperalgesia in SNI rats; however, MMP, which is a specific ERα blocker, cannot. Therefore, normal rats were administered with G1, PPT, and DPN, which are GPER-specific, ERα-specific, and ERβ-specific agonists, respectively. Only the GPER-specific agonist G1 could induce hyperalgesia to similar levels as that noted in the SNI model. Our findings suggest that GPER could be involved in SNI-induced neuropathic pain development; moreover, GPER is an important molecular component contributing to peripheral pain transduction. A study using a rat model of visceral pain demonstrated that spinal ERα mediates oestradiol-induced pronociception [51]. ERβ and ERα have specific advantages regarding analgesic effects [52]. Our findings suggest that GPER upregulation in the DRG, but not the SDH, may be an important component in SNI-induced neuropathic pain. It has also been reported that the DRG is the site of initial pain sensation, and the spinal cord mainly plays a role in conduction [8]. The roles of GPER downstream signalling pathways remain unclear. This study provided evidence regarding chronic pain by using the α2-GABAA receptor to evaluate pain behaviour and the role of GPER in pain. SNI allodynia is followed by downregulation of the α2-GABAA receptor, and these levels are improved by intrathecal G15 administration. Moreover, mechanical pain and cold hyperalgesia are alleviated. Additionally, we assessed the changes in the expression of other GABA subunits. The mRNA levels were consistent with the Western blotting results, indicating that GPER is involved in neuropathic pain by regulating α2-GABAA. Moreover, high α2-GABAA and GPER coexpression were noted on small and medium DRG neurons. Therefore, we will conduct follow-up research on GPER expression in the DRG.

We successfully established the rat SNI model and intrathecally administered GPER blockers. We examined the gene expression profiles with a focus on mRNA in neurons of SNI rats, sham rats, and SNI + G15 rats using RNA-Seq. We found several differentially expressed mRNAs. Furthermore, we validated their expression via a protein assay and examined the molecular functions, cellular components, and enriched biological processes of these DEGs by applying bioinformatics analysis. A sizeable proportion of these DEGs were exclusively involved in inflammatory processes; furthermore, IL-6 and IL-1β are of important significance. Injury to the peripheral sensory nerves causes a neuroinflammatory response in the somatosensory pathway, from the DRG to the spinal cord, which contributes to neuropathic pain [53]. Activating nociceptor sensory neurons through noxious stimuli triggers pain and increases capillary permeability and blood flow to yield neurogenic inflammation, which results from the antidromic activation of nociceptor peripheral terminals [54]. Additionally, mRNA enrichment analysis further identified the direct or indirect regulatory relationship that may exist in the GPER metabolic pathway under SNI conditions.

GPER upregulation is crucially involved in promoting neuroinflammation and DRG sensitization during chronic pain. We performed metabolomic analysis in neurons of rats in all three groups. Compared with the sham and SNI + G15 groups, the SNI group showed changes in β-alanine and nicotinic acid. Based on enrichment analysis, β-alanine exhibited the most significant changes through enrichment analysis among a series of metabolites. Furthermore, the administration of G15, which is a GPER blocker, reverses the SNI-induced increase in alanine and alleviates behavioural problems. A local intradermal injection of β-alanine directly induces itching in humans, which confirms that β-alanine induces itching through a peripheral, cutaneous mechanism [55, 56]. Additionally, certain mechanical stimuli mediated by recently discovered circuits contribute to the itch sensation in the spinal cord and brain. These circuits that mediate touch, pain, and itching could engage in cross modulation [57]. We administered β-alanine into the upper sheath of normal rats, and the behavioural test revealed that rats showed mechanical and cold hyperalgesia upon itching. After SNI, a continuous increase in β-alanine levels was noted in the rat DRG until 14 days. Western blot experiments also revealed an increased GPER levels, increased inflammation, decreased α2-GABAA levels, and excitation of DRG neurons after SNI. The high excitability state of DRG neurons after the G15 SNI model can be relieved. Moreover, there was enhanced excitability of DRG neurons was noted in normal rats after treatment with G1 and β-alanine. Supplementation with β-alanine has been shown to delay the accumulation of lactate during exercise by buffering the formation of lactate from pyruvate [58]. In this study, we selected small- and medium-sized neurons. Therefore, our findings demonstrate that the post-SNI GPER upregulation in small DRG neurons increases the levels of the inflammatory cytokines IL-1β and IL-6 and decreases α2-GABAA levels. In addition, β-alanine accumulation can be reversed through G15 administration. Both the secretion of inflammatory factors and the firing of neurons require energy consumption [59]. It is also possible that the energy generated during the continuous accumulation of β-alanine may cause the DRG cells to continuously produce inflammation and nerve excitability. β-Alanine can also positively regulate GPER formation similar to the cascade effect. TGR7 functions as a specific membrane receptor for β-alanine, which corresponds to MrgD. Mas-related GPCR D is specifically expressed in small-diameter nociceptive DRG neurons and is implicated in pain modulation [60]. We confirmed that β-alanine binds GPER, potentially explaining the chronicity of neuropathic pain. Notably, all male SD rats were used in this study, and the results also have certain limitations. It remains unclear whether the effect of oestrogen is the main contributor to sex differences in pain [61–63], However, as a sex hormone receptor, GPER is involved in the occurrence of pain, and the gender and content differences in the distribution in the body may also represent an important factor.

It is well known that the excitation of neurons is one of the main contributors to pain generation [64]. The main cell types in the DRG include neurons, microglia, and astrocytes, among which glial cells also play an important role in neuroinflammation [65]. The positive interaction between GPER and β-alanine produces neuroinflammation, and we wondered whether this effect also occurs in glial cells. To further clarify the location of the interaction, we detected GPER expression on neuronal cells and microglia but not on astrocytes. Thus, microglia also play an important role. We extracted primary neuronal cells and microglia from DRG cells and cultured them independently. β-Alanine-activated microglia and neurons release various proinflammatory and neurotoxic factors, such as IL-1β, IL-6, and iNOS, and downregulated GABAα2 levels. This finding was similar to the result of decreased neuronal excitability after in vivo administration of the blocking agent G15 after SNI in rats. After GPER protein expression was silenced by siRNA transfection, the abovementioned changes were no longer stimulated by β-alanine. Therefore, a positive interaction between GPER and β-alanine exists on both neurons and microglia in the DRG. Combined with the abovementioned neuronal excitation, the interaction has an amplifying effect on inflammation, and it has been reported that IL-1β expression in dopaminergic neurons is essential for the initiation and progression of PD. Thus, we boldly hypothesize that after the positive interaction between GPER and β-alanine, neurons are excited and secrete inflammatory substances with microglia, and inflammatory substances can also act on neurons to yield a positive feedback effect. This mechanism should be further explored. It is possible to locally deplete β-alanine and block GPER as a potentially effective clinical treatment for chronic neuropathic pain.

## Conclusions

In conclusion, we hypothesize that GPER activation in the neurons and microglia of the DRG causes a positive association between β-alanine and iNOS, IL-1β and IL-6 expression that ultimately results in the repression GABAα2 expression in post-SNI neuropathic pain and neuroinflammation development. Therefore, GPER blockers and β-alanine elimination may represent underlying molecular targets for controlling neuropathic pain without affecting normal sensation.

## Supplementary material

1. Transcriptomic raw date can be searched on https://www.ncbi.nlm.nih.gov/geo/query/acc.cgi?acc=GSE201163, accession number is GSE201163.

2. Metabolomic raw date can be searched on https://www.ebi.ac.uk/metabolights/editor/study/MTBLS4725/descriptors, accession number is MTBLS4725.

## Supplementary Information

**Additional file 1: Fig. S1**. (A) Intraplantar injection of the G protein-coupled oestrogen receptor-1 antagonist (G15; 1.8 μg/μl, 1 μl) into the subarachnoid space of SNI rats on day5. The mRNA content of oestrogen receptor-α, oestrogen receptor-β, and G protein-coupled oestrogen receptor and GABAα2 in SDH, there is no change except GABAα2, which was downregulated. (B) Western blot images for GPER, ERα, ERβ, and GABAα2 in the SDH. (C) There was no change in any protein in the spinal dorsal horn (SDH), except GABAα2, which was downregulated. Two-tailed Student’s t test. ^***^p < 0.001 versus SNI, ^###^*p* < 0.001 versus SNI, *n* = 3 in each group.

**Additional file 2: Table S1.** Numbers of Animals Used in the Different Experiments.

**Additional file 3: Table S2.** Primers Information of qPCR Experiment.

## Data Availability

The key data are contained in the figures, tables, and additional files. The datasets used and/or analyzed during this study can be further obtained from the corresponding author on reasonable request.

## Abbreviations

GPER: G protein-coupled oestrogen receptor
SDH: Spinal dorsal horn
DMSO: Dimethylsulfoxide
DRG: Dorsal root ganglion
ER: Oestrogen receptor
PWMT: Paw withdrawal mechanical threshold
PWCD: Paw withdrawal cold duration
PWTL: Paw withdrawal thermal latency
PBS: Phosphate-buffered saline
SNI: Spared nerve injury
RT-PCR: Reverse transcription-polymerase chain reaction
RNA-Seq: RNA sequencing
DEmRNA: Differentially expressed mRNA
qPCR: Real-time quantitative PCR
KEGG: Kyoto Encyclopedia of Genes and Genomes
GO: Gene ontology
DEG: Differentially expressed gene
iNOS: Inducible nitric oxide synthase
