# Supplementary material for: Positive interaction between GPER and β-alanine in the dorsal root ganglion uncovers potential mechanisms: mediating continuous neuronal sensitization and neuroinflammation responses in neuropathic pain
Source: J Neuroinflammation. 2022 Jun 21;19:164. doi: 10.1186/s12974-022-02524-9 (PMC9215054; doi:10.1186/s12974-022-02524-9)
Supplement: Supplementary file 2 — Additional file 2: Table S1. Numbers of Animals Used in the Different Experiments. [file 12974_2022_2524_MOESM2_ESM.docx]

| **Expriment** | **Simple size** | **Groups(n)** | **Sample（n）** | **Weight** |
| --- | --- | --- | --- | --- |
| **Behavioral test** | n=8 | 12 | 96 rat | 200-220 g |
| **Immunohistochemistry** | n=6 | 3 | 18 rat | 200-220 g |
| **Western blot** | n=4-8 | 7 | 36 rat | 200-220 g |
| **Patch-clamp recording** | n=4-6 neurons | 5 | 25 neurons/20 rats | 50–80 g |
| **Primary culture** | n=3 | 4 | 24 P1 rats | P1 |
| **Real-time polymerase**  **chain reaction** | n=4-8 | 3 | 18 rats | 200-220 g |
| **RNA-Seq** | n=6 | 3 | 18 rats | 200-220 g |
| **Metabolomics** | n=12 | 3 | 36 rats | 200-220 g |
| **β-Alanine Colorimetric Assay Kit** | n=3 | 11 | 33 rats | 200-220 g |
| **Total number of animals** |  |  | 275 rats | 200-220 g |
